# Baseline NT-ProBNP level predicts success of cardioversion of atrial fibrillation with flecainide

**DOI:** 10.1007/s12471-015-0659-8

**Published:** 2015-02-20

**Authors:** Ahmad Shoaib Amin, René H.J. Peters, Maaike Verstraaten, Arthur A.M. Wilde, Eugène M. Buijs

**Affiliations:** 1 Heart Center, Departments of Clinical and Experimental Cardiology, Academic Medical Center, University of Amsterdam, Meibergdreef 9, 1105 AZ Amsterdam, The Netherlands; 2 Department of Cardiology, Tergooi Hospitals, Rijksstraatweg 1, 1261 AN Blaricum, The Netherlands; 3 Department of Cardiology, Free University Medical Center, Amsterdam, The Netherlands

## Abstract

**Background:**

Patients with acute-onset symptomatic atrial fibrillation (AF) can be treated with flecainide. However, flecainide may induce arrhythmias and/or exaggerate heart failure. Therefore, validated markers to predict the efficacy of flecainide and prevent adverse effects are required. We hypothesised that lower NT-proBNP plasma levels correlate with higher success rates of cardioversion with flecainide in patients with AF.

**Methods:**

In this prospective single-centre study, we included 112 subsequent patients with acute-onset (< 24 h) symptomatic AF. Patients with symptoms of heart failure and ECG signs of ischaemia were excluded. Baseline laboratory measurements, including NT-proBNP, were done. Echocardiograms were performed ~ 2 weeks after restoration of SR.

**Results:**

Cardioversion with flecainide was successful in 91 patients (87 %). NT-proBNP was lower in patients with successful cardioversion (*P* < 0.001). Logistic regression indicated NT-proBNP as an independent predictor of successful cardioversion. A cut-off NT-proBNP value of 1550 pg/ml provided optimal test accuracy to predict successful cardioversion.

**Conclusion:**

In patients with < 24 h of symptomatic AF, NT-proBNP levels up to 1550 pg/ml correlate with high success rates (94 %) of cardioversion with flecainide. Conversely, NT-proBNP higher than 1550 pg/ml correlates with poor success rates (36 %). Further research is needed to validate the predictive value of NT-proBNP for successful cardioversion with flecainide.

## Introduction

Atrial fibrillation (AF) is one of the most common cardiac arrhythmias occurring in nearly 2 % of the general population [1]. Its prevalence is expected to increase in the future due to ageing [2, 3]. AF is associated with higher risk of death, heart failure, stroke, and hospitalisations and lower quality of life [4, 5]. In the aspect of AF therapy, rate control (i.e., restriction of the ventricular rate < 110 beats per minute) has been shown to be not inferior to rhythm control (i.e., restoring and maintaining sinus rhythm [SR]) in reducing the risk for mortality and morbidity and in improving the quality of life [6, 7]. However, patients with AF may be haemodynamically severely compromised in the acute setting or may remain symptomatic despite adequate rate control. In such cases, restoring SR is often (urgently) required [1].

SR can be achieved with direct current cardioversion (DCC) or antiarrhythmic drugs. DCC is an effective method of cardioversion with success rates of up to 94 %. However, it has potential disadvantages including the need for a fasting state and general anaesthesia and the risk of complications such as skin burns, and hypoxia and hypoventilation due to sedation [8]. Pharmacological cardioversion with antiarrhythmic drugs has the advantage of being simple, convenient and free of the need for a fasting state and anaesthesia. However, compared with DCC, success rates of pharmacological cardioversion are often remarkably lower [8, 9]. Moreover, antiarrhythmic drugs may induce arrhythmias such as bradycardia, sinus node arrest, atrioventricular block, and ventricular tachyarrhythmias through their proarrhythmic effects, and exaggerate (yet undiagnosed) heart failure through their negative inotropic effects [1, 8, 9]. Unfortunately, validated markers to predict the success of pharmacological cardioversion are lacking [10−13]. Identification of such markers may help clinicians to select their patients more adequately and, by doing so, increase success rates of pharmacological cardioversion and decrease the risk of serious adverse effects.

Plasma levels of B-type natriuretic peptide (BNP), and in particular its inactive N-terminal prohormone fragment (NT-proBNP), are reported to be elevated in AF and to rapidly decrease after restoration of SR [14−17]. However, contradictory data have been published on the role of NT-proBNP as a possible marker to predict the success of cardioversion [17−21]. Moreover, there are currently no data available on the use of plasma NT-proBNP level as a marker to predict the outcome of pharmacological cardioversion in patients with symptomatic AF. We therefore aimed to evaluate the predictive value of baseline plasma NT-proBNP level for successful cardioversion with flecainide in patients with < 24 h symptomatic AF (i.e., acute onset with AF symptoms lasting less than 24 h).

## Methods

### Study population and clinical data collection

In this prospective single-centre study, all adult patients (≥ 18 years of age) who presented to the cardiac emergency room of the Tergooi Hospitals between January 2011 and December 2012 with a primary diagnosis of AF were enrolled. Upon presentation, history was obtained, physical examination was performed, 12-lead electrocardiograms (ECGs) were taken, and baseline blood samples were drawn. If available, data on medical history were retrieved from hospital (electronic) patient records. Patients were excluded in the presence of one of the following criteria: permanent AF, symptoms lasting ≥ 24 h, supraventricular tachycardia other than AF on the ECG, haemodynamic instability, signs of heart failure, and ECG signs of acute or prior myocardial infarction, including ST-segment elevation, low QRS voltages, intraventricular conduction disturbances and/or pathological Q waves. Criteria used to detect these ECG changes were as described earlier by the Third Universal Definition of Myocardial Infarction (see reference 22). The study was approved by the institutional review committees and conforms to the principles outlined in the Declaration of Helsinki. The following clinical data were collected from all included patients: age, gender, smoking, medication use, history of thyroid disease, and CHA_2_DS_2_-VASc score (history of congestive heart failure, hypertension, diabetes, thromboembolic events, and vascular disease). The following data were collected from physical examination: heart rate, blood pressure, body weight and height, and whether clinical signs of congestive heart failure were present.

### Cardioversion

All included patients received intravenous flecainide under continuous haemodynamic and ECG monitoring during drug administration and for at least 6 h afterwards. Flecainide was administered in a dose of 2 mg per kg body weight (with a maximum dose of 150 mg) over 10 min. Patients in whom SR could not be obtained within 6 h after intravenous administration of flecainide underwent DCC. During these 6 h, patients were given nil per os instructions to reach a fasting state required for possible DCC. Patients who underwent DCC stayed under continuous monitoring for at least 3 h before they were allowed to leave the hospital.

### ECG analysis

Twelve-lead ECGs were made at baseline (upon presentation), during and after intravenous administration of flecainide, and after restoration of SR. If patients underwent DCC, 12-lead ECGs were performed before and immediately after the procedure. Twelve-lead ECGs were taken from all patients upon discharge. ECGs were analysed by individuals blinded to NT-proBNP levels and cardioversion outcome.

### Blood testing

Blood samples were drawn upon presentation and used to measure baseline plasma levels of haemoglobin, leukocytes, C-reactive protein, creatinine, glucose, NT-proBNP, and thyroid hormone profile. All laboratory measurements, including NT-proBNP, were performed by the hospital central laboratory according to the hospital standards and quality.

### Follow-up

Patients were scheduled for an outpatient follow-up appointment approximately 2 weeks after discharge, which included 12-lead ECG and echocardiography. Echocardiograms were used to measure left ventricular function and left atrial diameter. ECGs and echocardiograms were analysed by individuals blinded to NT-proBNP levels and cardioversion outcome.

### Statistics

Categorical variables are presented as frequencies and percentages and were compared by χ^2^ test or Fisher’s exact test, where appropriate. Continuous variables are presented as means with standard error (SEM) if normally distributed or as medians with interquartile range (IQR) if otherwise, and were compared by unpaired *t* tests or the Mann−Whitney U-test, respectively. Because of the skewed distribution of NT-proBNP, logarithmic transform (log NT-proBNP) was computed to approach a normal distribution, which was confirmed by the Kolmogorov−Smirnov test. The independent effect of multiple variables on outcome of cardioversion with flecainide and on plasma NT-proBNP levels was tested using univariate analysis. All variables with a *P* < 0.20 at univariate analysis were selected for multivariate logistic regression analysis. The diagnostic utility of plasma NT-proBNP for predicting the outcome of cardioversion with flecainide was assessed by obtaining a receiver operating characteristic (ROC) curve. Statistical significance was defined as *P* < 0.05. Statistical analyses were performed using SPSS (Chicago, Illinois, USA).

## Results

### Study population

In total, 112 patients met the inclusion criteria and underwent pharmacological cardioversion with intravenous flecainide. Cardioversion with flecainide was successful in 97 patients with < 24 h of symptomatic AF (87 %). In all the remaining 15 patients with < 24 h of symptomatic AF (13 %), in whom SR could not be obtained within 6 h after intravenous infusion of flecainide, SR could be achieved with DCC.

### Baseline characteristics

**Figure 1:**
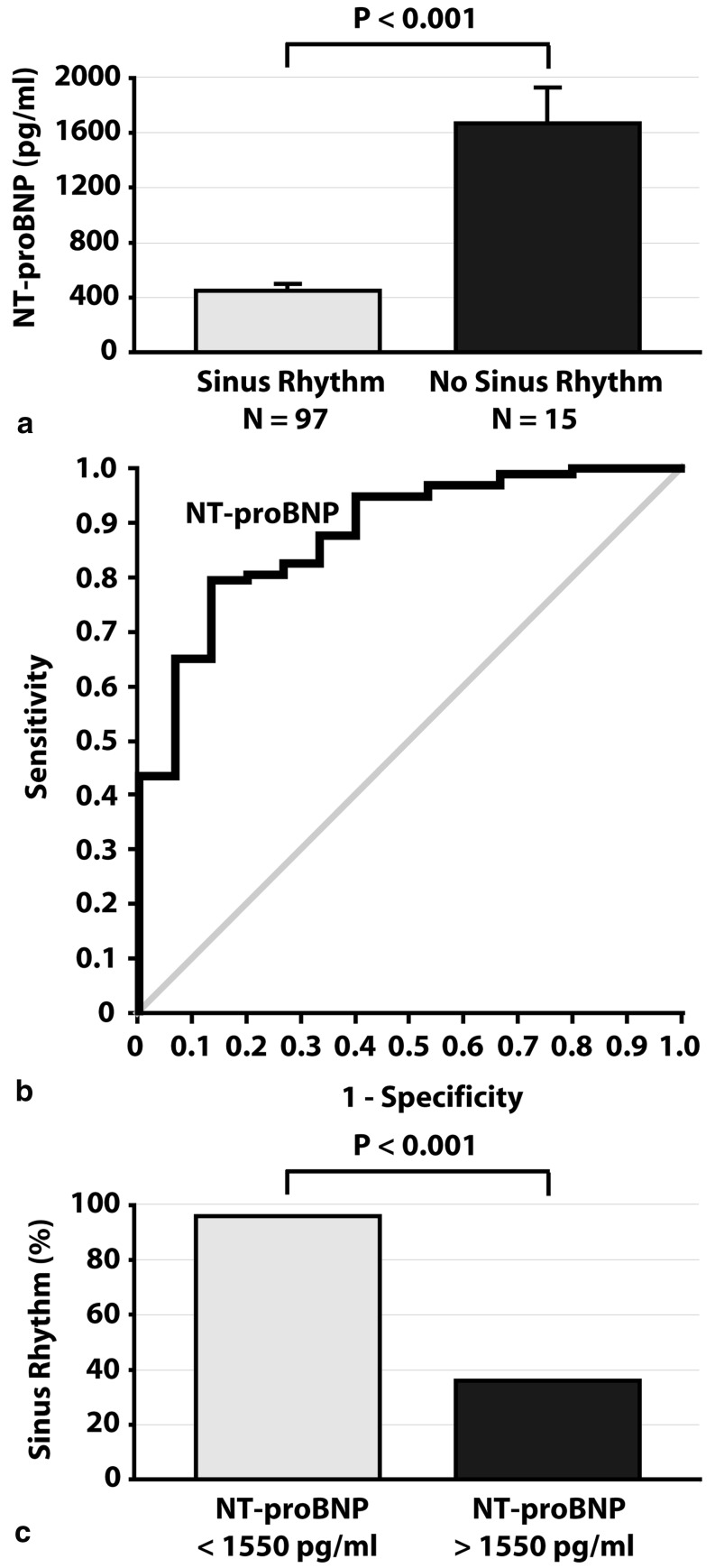
Panel **a** displays baseline plasma NT-proBNP levels (upon admission) during atrial fibrillation in patients who converted to sinus rhythm (SR) versus those who did not convert to SR after intravenous administration of flecainide. N indicates number of patients. Panel **b** displays the receiver operating curve of plasma NT-pro-BNP levels as a predictor of outcome of cardioversion with intravenous flecainide. Panel **c** displays the proportion of patients who converted to SR after intravenous administration of flecainide who had baseline plasma NT-proBNP levels lower than 1550 pg/ml or higher than 1550 pg/ml

Patient characteristics at baseline (before cardioversion) are displayed in Table 1. Age, proportion of men, body mass index, CHA_2_DS_2_-VASc score, drug use, and the prevalence of common conditions associated with AF (e.g., coronary artery disease, hypertension, diabetes, thyroid disease, and smoking) did not differ between patients with successful cardioversion with flecainide and patients in whom SR could not be obtained with flecainide. However, baseline plasma NT-proBNP level was significantly lower in patients who converted to SR than in patients in whom SR could not be obtained with flecainide (*P* < 0.001; Table 1 and Fig. 1a). Left atrial diameter was significantly larger in patients who did not convert to SR than patients who obtained SR after flecainide (*P* = 0.019). The logistic regression model included the following patient characteristics as covariates (*P* < 0.20 at univariate analysis): age, heart rate, history of thyroid disease, left atrial diameter and log NT-proBNP. Logistic regression analysis indicated log NT-proBNP as the only independent predictor of cardioversion outcome with flecainide (*P* = 0.002). Left atrial diameter did not reach statistical significance as an independent predictor of cardioversion outcome (*P* = 0.078).

**Table 1:** Baseline characteristics of the study population at baseline according to the outcome of cardioversion with intravenous flecainide

	All patients	Conversion to SR after flecainide	Persistence of AF after flecainide^a^	*p* value
	(*n = 112*)	(*n = 97*)	(*n = 15*)	
**Baseline**				
Age (*years*)	63 ± 1	62 ± 1	67 ± 3	0.151
Men (*n, %*)	60 (54)	50 (52)	10 (67)	0.415
Heart rate (*beats/minute*)	125 ± 2	126 ± 2	115 ± 7	0.147
Systolic blood pressure (*mmHg*)	139 ± 2	140 ± 2	134 ± 5	0.288
Diastolic blood pressure (*mmHg*)	86 ± 1	86 ± 2	85 ± 3	0.762
BMI > 25 kg/m^2^ (*n, %*)	31 (28)	25 (26)	6 (40)	0.403
AF *de novo* (*n, %*)	59 (53)	51 (53)	8 (53)	0.884
Coronary artery disease (*n, %*)	11 (10)	9 (9)	2 (13)	0.980
Hypertension (*n, %*)	52 (46)	47 (48)	5 (33)	0.415
Diabetes (*n, %*)	8 (7)	7 (7)	1 (7)	0.644
Thyroid disease	5 (4)	3 (3)	2 (13)	0.265
Current smoking (*n, %*)	24 (21)	21 (22)	3 (20)	0.847
Smoking history (*n, %*)	27 (24)	22 (23)	5 (33)	0.566
CHA_2_DS_2_-VASc	2 (1–3)	2 (1–3)	2 (0.25–3)	0.815
CHA_2_DS_2_-VASc ≥ 1 (*n, %*)	88 (79)^b^	77 (79)^b^	11 (73)	0.847
CHA_2_DS_2_-VASc ≥ 1 & AF *de novo* (*n, %*)	44 (39)	40 (41)	4 (27)	0.429
Oral anticoagulation use (*n, %*)	39 (35)	32 (33)	7 (47)	0.457
Class I or III antiarrhythmic drugs (*n, %*)	18 (16)	14 (14)	4 (27)	0.411
Class II or IV antiarrhythmic drugs (*n, %*)	16 (14)	15 (15)	1 (7)	0.610
Haemoglobin (*mmol/l*)	9.2 ± 0.1	9.1 ± 0.1	9.2 ± 0.2	0.689
Leukocytes (*× 10* ^*9*^ */l*)	7.5 ± 0.2	7.5 ± 0.2	7.9 ± 0.4	0.338
C-reactive protein (*mg/l*)	5.4 ± 1.2	5.4 ± 1.3	5.5 ± 3.2	0.984
Normal glomerular filtration rate (*n, %*)	110 (98)	95 (98)	15 (100)	0.627
Glucose (*mmol/l*)	6.2 ± 0.1	6.3 ± 0.1	5.9 ± 0.4	0.213
Thyroid-stimulating hormone (*mE/l*)	2.4 ± 0.2	2.4 ± 0.2	3.2 ± 0.7	0.955
NT-ProBNP (*pg/ml*)*,* mean (SEM)	612 ± 69	449 ± 52	1669 ± 263	< 0.001
NT-ProBNP (*pg/ml*)*,* median (IQR)	316 [111–808]	266 [101–529]	1597 [779–2430]	< 0.001
**Follow-up**				
SR at follow-up (*n, %*)	96 (86)	81 (84)	15 (100)	0.193
Left atrial size (*mm*)	40 ± 1	39 ± 1	43 ± 2	0.019
Left ventricular ejection fraction > 55 % (*n*)	112 (100 %)	97 (100 %)	15 (100 %)	1.000

*N* indicates number of patients. Data are presented as means ± standard error (SEM) or medians with interquartile range (IQR)

*AF* atrial fibrillation, *SR* sinus rhythm, *BMI* body mass index

^a^ In all 15 patients in whom SR could not be obtained after intravenous infusion of flecainide, SR was achieved with direct current cardioversion (DCC)

^b^ Five patients within this group with CHA_2_DS_2_-VASc ≥ 1 were female patients with gender as the only risk factor for stroke

Figure 1b shows the ROC curve for baseline plasma NT-proBNP levels as a predictor of outcome after cardioversion with flecainide. The area under the curve was 0.8797 (95 % confidence interval 07932–0.9663; *P* < 0.001). An analysis of cut-offs of baseline plasma NT-pro-BNP levels was carried-out. NT-proBNP levels lower than 1550 pg/ml had a sensitivity of 95 % and a specificity of 60 %, and provided optimal predictive values for the outcome of cardioversion. In our study population, 98 patients had plasma NT-proBNP levels lower than 1550 pg/ml. Of these patients, 92 (94 %) converted to SR with flecainide. These data indicate that plasma NT-proBNP levels lower than 1550 pg/ml correlate with a success rate of 94 % for cardioversion with flecainide (i.e., positive predictive value of 94 %). Conversely, 14 patients had plasma NT-proBNP levels higher than 1550 pg/ml, of whom only five (36 %) converted to SR with flecainide (i.e., negative predictive value of 64 %).

### Medication use before and after cardioversion

At baseline, use of antiarrhythmic drugs was not different between patients with successful cardioversion with flecainide and patients in whom SR could not obtained with flecainide (Table 1). The antiarrhythmic drug regimen was not changed after cardioversion or at discharge. At baseline, oral anticoagulation therapy had already been initiated in patients with prior episodes of AF and CHA_2_DS_2_-VASc ≥ 1 (Table 1), except for female patients with gender as the only risk factor for stroke (*n* = 5). In all patients with AF *de novo* and CHA_2_DS_2_-VASc ≥ 1 (*n* = 44), oral anticoagulation therapy was initiated at the time of hospital discharge (Table 1).

### Discharge and follow-up

No clinical adverse effects and arrhythmias that could be related to flecainide were observed during monitoring after cardioversion. All patients had SR at the time of hospital discharge. At follow-up 2 weeks after hospital discharge, 96 patients still had SR (86 %), while AF had recurred in the remaining 16 patients (14 %) (Table 1). Out of the 97 patients with successful cardioversion with flecainide, 81 (84 %) still had SR at follow-up while in 16 patients (14 %) AF had recurred. All 15 patients in whom SR could not be obtained with flecainide and who had undergone successful DCC had SR at follow-up. Characteristics of patients according to their heart rhythm at follow-up (SR or AF; regardless of the outcome of cardioversion with flecainide) are shown in Table 2. Characteristics of patients according to the outcome of cardioversion with flecainide and heart rhythm at follow-up (SR or AF) are shown in Table 3. Patient characteristics, including baseline plasma NT-proBNP level and left atrial diameter, did not differ between patients who had SR at follow-up and those in whom AF had recurred at follow-up (regardless of the outcome of cardioversion with flecainide; Table 2). Patient characteristics, including baseline plasma NT-proBNP level and left atrial diameter, also did not differ between patients with successful cardioversion with flecainide who still had SR and patients with successful cardioversion with flecainide in whom AF had recurred at follow-up (Table 3). No thromboembolic complications were found in any of the patients at follow-up.

**Table 2:** Characteristics of the study population according to the heart rhythm at follow-up (and regardless of the outcome of cardioversion with intravenous flecainide)

	SR at follow-up	AF at follow-up	*p* value
	(*n = 96*)	(*n = 16*)	
Conversion to SR with flecainide i.v.	81 (84)	16 (100)	0.193
Persistence of AF after flecainide i.v.	15 (16)	0 (100)	0.193
Age (*years*)	62 ± 7	66 ± 13	0.274
Men (*n, %*)	50 (52)	10 (63)	0.727
BMI > 25 kg/m^2^ (*n, %*)	29 (30)	2 (13)	0.244
AF *de novo* (*n, %*)	53 (55)	6 (38)	0.297
Coronary artery disease (*n, %*)	10 (10)	1 (6)	0.948
Hypertension (*n, %*)	45 (47)	7 (44)	0.969
Diabetes (*n, %*)	7 (7)	1 (6)	0.708
Thyroid disease	4 (4)	1 (6)	0.779
Current smoking (*n, %*)	22 (23)	2 (13)	0.541
Smoking history (*n, %*)	26 (27)	1 (6)	0.137
CHA_2_DS_2_-VASc	2 [1.0–3.0]	1.5 [1–2.5]	0.997
CHA_2_DS_2_-VASc ≥ 1 (*n, %*)^a^	75 (78)	13 (81)	0.963
Oral anticoagulation use (*n, %*)	32 (33)	7 (44)	0.599
Class I or III antiarrhythmic drugs (*n, %*)	15 (16)	3 (19)	0.958
Class II or IV antiarrhythmic drugs (*n, %*)	15 (16)	1 (6)	0.544
NT-ProBNP (*pg/ml*)*,* mean (SEM)	637 ± 78	465 ± 109	0.384
NT-ProBNP (*pg/ml*)*,* median (IQR)	316 [108–840]	344 [151–693]	0.940
Left atrial size (*mm*)	39 ± 0	40 ± 1	0.801
Left ventricular ejection fraction > 55 % (*n*)	96 (100 %)	16 (100)	1.000

*N* indicates number of patients. Data are presented as means ± standard error (SEM) or medians with interquartile range (IQR)

**Table 3:** Characteristics of the study population according to the outcome of cardioversion with intravenous flecainide and according to the heart rhythm at follow-up

	Conversion to SR after flecainide SR at follow-up	Conversion to SR after flecainide AF at follow-up	*p* value	Persistence of AF after flecainide^a^ SR at follow-up
	(*n = 81*)	(*n = 16*)		(*n = 15*)
Age (*years*)	62 ± 1	66 ± 3	0.206	67 ± 3
Men (*n, %*)	40 (49)	10 (63)	0.493	10 (67)
BMI > 25 kg/m^2^ (*n, %*)	23 (28)	2 (13)	0.227	6 (40)
AF *de novo* (*n, %*)	45 (56)	6 (38)	0.259	8 (53)
Coronary artery disease (*n, %*)	8 (10)	1 (6)	1.000	2 (13)
Hypertension (*n, %*)	40 (49)	7 (44)	0.890	5 (33)
Diabetes (*n, %*)	6 (7)	1 (6)	1.000	1 (7)
Thyroid disease	2 (2)	1 (6)	0.421	2 (13)
Current smoking (*n, %*)	19 (23)	2 (13)	0.510	3 (20)
Smoking history (*n, %*)	21 (26)	1 (6)	0.109	5 (33)
CHA_2_DS_2_-VASc	2 [1–3]	1.5 [1–2.5]	0.981	2 (0.25–3)
CHA_2_DS_2_-VASc ≥ 1 (*n, %*)^*a*^	64 (79)^b^	13 (81)	1.000	11 (73)
Oral anticoagulation use (*n, %*)	25 (31)	7 (44)	0.477	7 (47)
Class I or III antiarrhythmic drugs (*n, %*)	11 (14)	3 (19)	0.697	4 (27)
Class II or IV antiarrhythmic drugs (*n, %*)	14 (17)	1 (6)	0.453	1 (7)
NT-ProBNP (*pg/ml*)*,* mean (SEM)	445 ± 58	465 ± 109	0.891	1669 ± 263
NT-ProBNP (*pg/ml*)*,* median (IQR)	266 [94–510]	344 [152–693]	0.443	1597 (779–2430)
Left atrial size (*mm*)	39 ± 1	40 ± 1	0.546	43 ± 2
Left ventricular ejection fraction > 55 % (*n*)	81 (100)	16 (100)	1.000	15 (100 %)

N indicates number of patients. Data are presented as means ± standard error (SEM) or medians with interquartile range (IQR)

*AF* atrial fibrillation, *SR* sinus rhythm, *BMI* body mass index

^a^ In all 15 patients in whom SR could not be obtained after intravenous infusion of flecainide, SR was achieved with direct current cardioversion (DCC)

^b^ Five patients within this group with CHA_2_DS_2_-VASc ≥ 1 were female patients with gender as the only risk factor for stroke. P values indicate statistical difference between patients with successful cardioversion with flecainide who still had SR at follow-up and those with successful cardioversion with flecainide in whom AF had recurred

## Discussion

In this study, we aimed to evaluate the predictive value of baseline plasma NT-proBNP level for successful cardioversion with flecainide in patients with < 24 h symptomatic AF. In 112 patients with AF and AF-related symptoms lasting less than 24 h, we found that: (1) patients who obtained SR after intravenous flecainide had significantly lower baseline plasma NT-proBNP levels than patients in whom SR could not be obtained with flecainide, (2) baseline plasma NT-proBNP level is an independent predictor of immediate cardioversion outcome with intravenous flecainide, (3) baseline plasma NT-ProBNP levels lower than 1550 pg/ml correlate with a high cardioversion success rate (94 %), (4) cardioversion with flecainide has a poor success rate (36 %) in AF patients with baseline NT-ProBNP levels higher than 1550 pg/ml, and (5) baseline plasma NT-ProBNP levels were not associated with maintenance of SR 2 weeks after successful cardioversion. We also found that left atrial diameter was significantly larger in patients in whom SR could not be obtained with flecainide than in patients with SR after intravenous administration of flecainide. However, left atrial diameter was not indicated as an independent predictor of cardioversion outcome by logistic regression analysis. Our data provide evidence that baseline plasma NT-proBNP level may be used as an easy, effective and safe marker to predict the immediate outcome of cardioversion with flecainide in patients with acute-onset < 24 h symptomatic AF. This may help to increase the cardioversion success rates and decrease the risk of serious adverse effects. However, our data also suggest that baseline plasma NT-proBNP levels do not predict long-term maintenance of SR after cardioversion.

Compared with DCC, pharmacological cardioversion has the advantage of being simple, convenient and free of the need for a fasting state and general anaesthesia. Unfortunately, success rates of pharmacological cardioversion are lower than for DCC, ranging between 50 and 80 % [23]. In addition, antiarrhythmic drugs that are used for pharmacological cardioversion may exaggerate heart failure and increase the risk for arrhythmias such as bradycardia, sinus node arrest, atrioventricular block, and ventricular tachyarrhythmias [1, 8, 9]. Several factors have been associated with higher success rates of cardioversion (electrically or pharmacologically), including younger age, absence of structural heart disease, smaller left atrial diameter, normal left ventricular function, shorter AF duration, use of class I or III antiarrhythmic drugs, and lower plasma levels of C-reactive protein [10−13]. However, as was confirmed by this study, none of these factors has been shown as a reliable and useful predictor of successful cardioversion.

BNP and NT-proBNP are produced in both the atria and the ventricles. Their release is mainly stimulated by myocardial wall stress. Plasma levels of BNP and NT-proBNP are reported to be elevated in AF, to predict occurrence of AF *de novo* and to decrease after restoration of SR [14−17, 24]. This elevation is speculated to result from atrial stretch due to atrial overload during AF. Contradictory data have been published regarding the role of plasma BNP and NT-proBNP levels as predictors of successful cardioversion [17−21]. However, most of the previous studies were performed in a small number of patients with variable duration of AF (days to months) in whom structural heart diseases were excluded in advance. In our study, we evaluated the predictive value of plasma NT-proBNP for successful cardioversion in a setting that closely resembles the daily clinical practice. To do so, we performed a study in patients who sought medical help for acute-onset AF-related symptoms by presenting to the cardiac emergency room. We included patients with symptoms lasting less than 24 h using flecainide for cardioversion because of prior evidence of its greater efficacy to restore SR compared with other antiarrhythmic agents [23]. Probably, by using proper inclusion and exclusion criteria, we did not observe any serious adverse effects that could be related to flecainide. A high success rate of cardioversion (87 %) was therefore achieved. By using a cut-off value of 1550 pg/ml for baseline plasma NT-proBNP, even a higher success rate of 94 % could be achieved, which is similar to success rates reported for DCC.

Flecainide is a class 1C antiarrhythmic drug which possesses the ability to block the cardiac sodium channel. Flecainide may also facilitate arrhythmias by slowing conduction of electrical stimuli through the heart [25, 26]. In patients with AF, the slowing of electrical conduction may be reflected as prolongation of QRS interval duration during flecainide infusion. In our study, patients with prolonged QRS durations at baseline were excluded, and marked QRS interval prolongation or arrhythmias during and after flecainide infusion were not observed during continuous ECG monitoring. However, we did not measure QRS interval durations at different time points during flecainide infusion, and thus cannot exclude subtle flecainide-induced QRS changes in our patients. In addition, it must be noted that all patients in our study had normal left ventricular function, and it remains to be investigated whether a NT-proBNP cut-off value of 1550 pg/ml may also be used in patients with impaired left ventricular function, a condition that is also associated with elevated NT-proBNP levels.

In conclusion, this prospective single-centre study in patients with acute-onset (< 24 h) symptomatic AF, and no manifest signs of heart failure and cardiac ischaemia, showed that baseline plasma NT-ProBNP levels up to 1550 pg/ml correlate with high success rates of cardioversion with flecainide. Conversely, cardioversion with flecainide had a poor success rate in patients with NT-ProBNP levels higher than 1550 pg/ml. Therefore, cardioversion with flecainide may be a highly effective and safe therapy in patients with acute-onset (< 24 h) symptomatic AF and baseline plasma NT-proBNP levels lower than 1550 pg/ml. Moreover, our data suggest that the indication for flecainide use for cardioversion in patients with < 24 h symptomatic AF and NT-ProBNP levels higher than 1550 pg/ml may be reconsidered. In addition, our data also suggest that baseline NT-proBNP levels may not predict long-term maintenance of SR after successful cardioversion. Further prospective studies in patients with AF are needed to validate our finding concerning the predictive value of baseline plasma NT-proBNP levels for successful cardioversion with flecainide in patients with acute-onset symptomatic AF.

### Source of funding

The Netherlands Heart Foundation.

### Conflict of interest

None.
